# Autoimmune Glial Fibrillary Acidic Protein Astrocytopathy: The Role of a Neurourogist From the Intensive Care Unit to Improving the Patient’s Quality of Life

**DOI:** 10.7759/cureus.67903

**Published:** 2024-08-27

**Authors:** Ioannis Tsikopoulos, Georgios Antoniadis, Vasileios Sakalis, Stamatios Katsimperis, Michalis Samarinas

**Affiliations:** 1 Neurourology, Royal National Orthopaedic Hospital, London, GBR; 2 Urology, General Hospital of Larissa, Larissa, GRC; 3 Urology, Hippokration Hospital of Thessaloniki, Thessaloniki, GRC; 4 Urology, National and Kapodistrian University of Athens, Athens, GRC; 5 Urology, Sismanogleio General Hospital, Athens, GRC

**Keywords:** neurourology, minimally invasive surgeries, botox injections, multidisciplinary treatments, urodynamic studies, urology services

## Abstract

Autoimmune glial fibrillary acidic protein (GFAP) astrocytopathy is an autoimmune disease that involves GFAP autoantibodies in the cerebrospinal fluid (CSF) and serum. Clinical manifestations exhibit diverse symptoms affecting various brain regions and spinal cord. Diagnosis challenges persist due to the absence of standardized criteria, yet steroid therapy shows promise despite varied responses.

Herein we present the case of a 30-year-old male with meningoencephalomyelitis symptoms, later diagnosed with autoimmune astrocytopathy. Treatment involved plasmapheresis, corticosteroids, and mycophenolate mofetil, with a positive outcome.

Neurourological complications, including acute urinary retention, prompted catheterization, and urodynamic studies revealed detrusor overactivity. Timely intervention enabled the restoration of bladder function, underscoring the importance of specialized care in complex neurologic conditions for improving patients' quality of life (QoL). This case emphasizes the significance of early neurourological intervention and the role of specialized centers in delivering tailored care for better patient outcomes.

## Introduction

Autoimmune glial fibrillary acidic protein (GFAP) astrocytopathy is an autoimmune nervous system disorder established only in 2016 that involves the presence of GFAP autoantibodies, particularly IgG targeting GFAPα, detected in both cerebrospinal fluid (CSF) and serum of affected individuals. Importantly, while GFAP antibodies serve as a biomarker for immune inflammation, they do not directly induce pathological changes. The pathology of GFAP astrocytopathy demonstrates considerable heterogeneity in affected human populations, commonly affecting individuals aged over 40, typically displaying acute or subacute onset. Clinical symptoms include fever, headaches, encephalopathy, involuntary movement, myelitis, and visual abnormalities. Lesions often affect various brain regions, including subcortical white matter, basal ganglia, hypothalamus, brainstem, cerebellum, and spinal cord. A distinct MRI feature involves radial gadolinium enhancement in the brain's white matter, appearing linear and perivascular, perpendicular to the ventricle. Currently, there are no universally agreed-upon diagnostic criteria for GFAP astrocytopathy, and coexisting neural autoantibodies complicate its diagnosis [1]. Establishing a standard treatment approach remains elusive, although steroid therapy shows positive responses in many patients, some are prone to relapses or severe outcomes, highlighting the complexity and variability of this condition's management [2].

Neurogenic lower urinary tract dysfunction (NULTD) often emerges as a consequence of neurological impairment at various levels of the central nervous system [3]. However, in cases where extensive neurological damage exists, the subsequent impact on the lower urinary tract presents a spectrum of multifaceted symptoms that challenge the efficacy of traditional urological interventions. Our specific case involved the identification of a neurogenic bladder condition attributed to a posterior cerebral disease, specifically autoimmune astrocytopathy. This complex neurological disorder resulted in a series of urinary dysfunctions, underscoring the intricate interplay between central nervous system pathology and its profound influence on lower urinary tract functionality.

## Case presentation

A 30-year-old male with an unremarkable medical history presented to the ER department with a high fever (39.5^o^C) and meningoencephalomyelitis-associated symptoms for five days. His mental status remained lucid, and a comprehensive physical examination revealed no irregularities. However, a neurological assessment exhibited stiffness in the neck and a positive Kernig's sign. The initial laboratory investigations, CSF culture, and CT scan showed no abnormalities. However, due to a decline in consciousness level (Glasgow Coma Scale (GCS) 10/15), a second lumbar puncture was performed, revealing increased white blood cells (355/μl), elevated total protein (222 mg/dL), and reduced glucose (52 mg/dL) in the CSF (Table 1).

**Table 1:** Cerebrospinal fluid values

Cerebrospinal fluid	Second puncture values	Normal values
White blood cells	355/μL	0-5/µL
Total protein	222mg/mL	15-45mg/mL
Glucose	52mg/dL	50-80mg/dL

**Figure 1:**
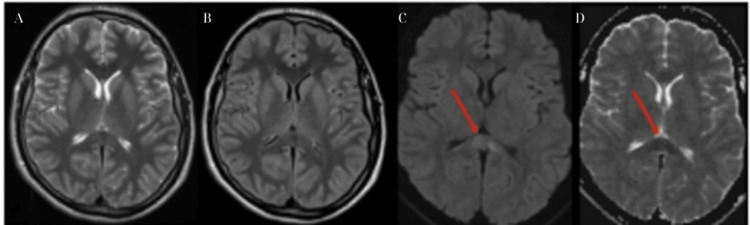
Initial MRI imaging: (A) initial T2 sequence appeared normal; (B) meningeal and linear-perivascular enhancements were observed in the T1 sequences following gadolinium administration; (C) signal increase in DWI; (D) decrease in the ADC map indicated restricted water in the splenium of the corpus callosum, indicative of cytotoxic edema (red arrows). DWI: diffusion-weighted imaging; ADC: apparent diffusion coefficient

Consequently, the patient was transferred to the ICU as the condition worsened (GCS 6/15). Despite extensive investigation ruling out infectious and autoimmune diseases, bone marrow biopsy, and CSF cytology, elevated levels of anti-GFAP antibodies were detected early in the disease and decreased during remission, as observed using a cell-based assay. Notably, heightened CSF levels of GFAP protein (CSF-enzyme-linked immunosorbent assay (ELISA)) were noted during the active phase and declined to negligible levels seven months post-disease onset. Serum levels of neurofilament light (NfL) (measured by the SIngle MOlecule Array (SIMOA)) were notably elevated across all measurements, peaking during the active phase (day 38) and decreasing during baseline and remission (day 80). The initial brain MRI revealed meningeal and linear perivascular enhancement post-gadolinium administration, along with changes in the corpus callosum (Figures 1A-1D).

Fortunately, he was responsive to targeted treatment (five plasmapheresis cycles followed by IV methylprednisolone and additional mycophenolate-mofetil), and as a result, he was extubated after two weeks. Screening for malignancies through CT and ultrasound showed negative results.

In means of urology care, a few days post admission the patient developed acute urinary retention necessitating urinary catheterization. As a result, while inpatient at the ICU, neurourological consultation was requested, and after evaluation, the removal of the indwelling catheter was advised along with the empirical initiation of fesoterodine 8 mg and gradual introduction to clean intermittent catheterizations (CIC) five to six times/day in the ICU. Furthermore, since the patient was alert and able to communicate, he was scheduled for urodynamics (UDS), considering all the possible artifacts associated with an examination with technical difficulties.

**Figure 2:**
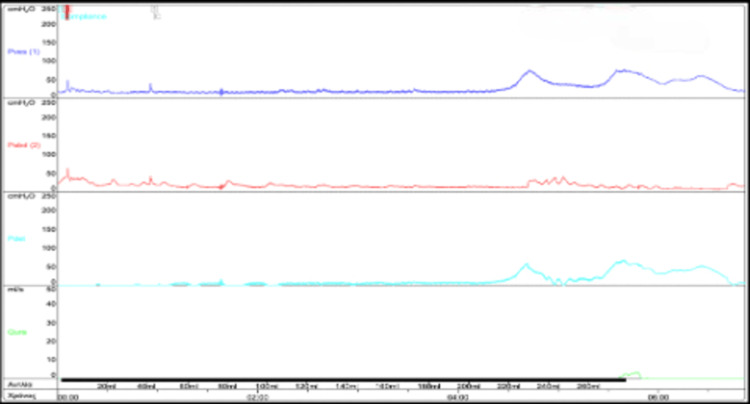
Baseline pressure flow study of the patient with an EDSS score of nine and on fesoterodine 8 mg; during the voiding phase, a micturition followed a terminal DO with high pressure (50 cmH2O). EDSS: Expanded Disability Status Scale; DO: detrusor overactivity

The patient underwent UDS in a supine position under the standards of the International Continence Society (ICS) in our neurourology clinic. The filling cystomanometry showed phasal and terminal detrusor overactivity (DO) of high pressures (30 cmH_2_O) without urine leakage, with non-specific sensation, low bladder compliance, and limited bladder capacity (150 ml). No signs of autonomic dysreflexia were observed. During the voiding phase, a micturition followed a terminal DO with high pressure (50 cmH_2_O) and a post-void residual (PVR) of 100 ml, while flow rates could not be evaluated due to the patient’s position (Figure 2).

**Figure 3:**
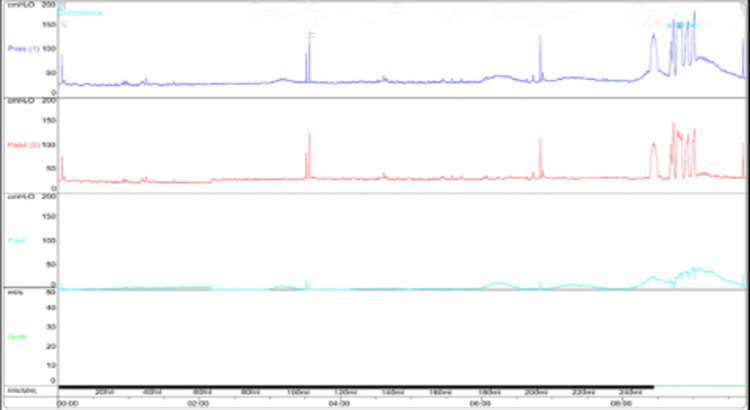
Pressure flow study of the patient with an EDSS score of seven, three months after the initial study; phasal and low-pressure DO, bladder capacity of 280 ml, and normal compliance were recorded. EDSS: Expanded Disability Status Scale; DO: detrusor overactivity

He was rescheduled for UDS as an outpatient with an Expanded Disability Status Scale (EDSS) score of seven, three months later. We documented only phasal and low-pressure DO, bladder capacity of 280 ml and normal compliance. However, he complained about urine leakage, especially when CIC voiding exceeded 250 ml (Figure 3).

**Figure 4:**
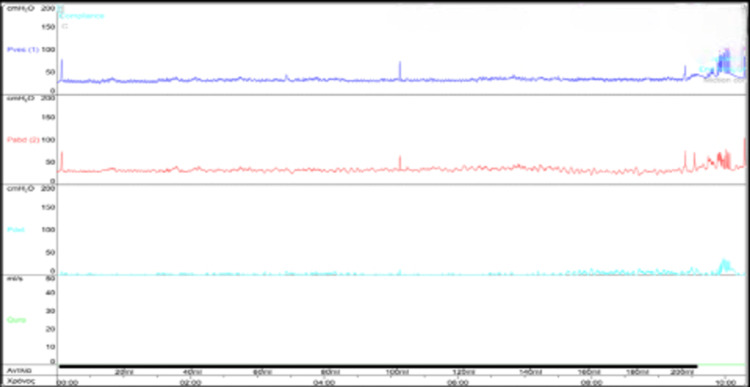
Pressure flow study, four weeks after intradetrusor BOTOX-A intervention and an EDSS score of seven; absence of DO was noted EDSS: Expanded Disability Status Scale; DO: detrusor overactivity

We suggested 200 units of intradetrusor botulinum A (ID-BOTOX) injections as an off-label treatment, and after the patient’s consent, we proceeded with the intervention. Four weeks later, the urodynamic study revealed an absence of DO and neither voiding nor urine leakage with a bladder capacity of 400 ml (Figure 4). The patient continued on CIC, five to six times/day after his ID-BOTOX session.

When the patient’s EDSS score improved to five, on his follow-up appointment the patient was happy with his bladder management as he was now dry and asked for his fertility status along with available options. After a detailed consultation, in the presence of his wife, micro-testicular sperm extraction (TESE) was advised, and pregnancy was finally achieved with in vitro fertilization.

## Discussion

Autoimmune GFAP astrocytopathy was officially recognized as an autoimmune disorder of the nervous system as recently as 2016. Fang et al. were the first to describe human GFAP astrocytopathy in 2016. This paper highlighted the identification of GFAP-IgG antibodies discovered in either the serum or CSF, showing specificity toward a cytosolic intermediate filament protein present in astrocytes [2]. The CSF of patients with autoimmune GFAP, according to the literature, has shown a notably higher positive predictive value for GFAP antibodies compared to serum. Two recommended methods for GFAP antibody detection are tissue-based assays (TBA) and cell-based assays (CBA) [1]. In a retrospective analysis of 102 patients who tested positive for GFAP-IgG, Flanagan et al. investigated several key aspects: the specificity of serum and CSF testing, evaluation of the clinical and radiological phenotype, the importance of concurrent antibodies, and the responses to therapy. The findings highlighted that CSF-GFAPαa-IgG exhibits high specificity for an autoimmune disorder affecting the central nervous system (CNS) that responds well to immunotherapy [3]. In that same year, Yang et al. conducted a study evaluating treatment responses in seven individuals positive for GFAP-IgG, tracking their progress over the long term. Despite the administration of steroids and immunosuppressive agents, certain patients with GFAP astrocytopathy exhibited an inadequate response to treatment [4]. Additionally, Long et al., in a separate study, outlined the clinical, radiological, and pathological characteristics observed in 19 individuals positive for CSF-GFAP-IgG. The neuropathological and immunopathological features identified in GFAP astrocytopathies comprised perivascular inflammation, as well as the depletion of astrocytes and neurons [5]. Another study conducted in the USA confirmed CSF GFAPα-IgG as a specific marker for autoimmune meningoencephalomyelitis, showing a good response to corticosteroids. For unresponsive cases, it suggests checking for concurrent NMDA-R-IgG or malignancy. Additionally, GFAP-IgG myelitis exhibits distinct spinal cord lesions, often longitudinally extensive, centrally located, and showing subtle imaging features compared to AQP4-IgG lesions [6].

In this specific medical case, the patient demonstrated a favorable response to the administration of corticosteroids, a therapeutic intervention that proved instrumental in averting the immediate life-threatening risks. The multifaceted treatment approach adopted encompassed a multidisciplinary strategy aimed at addressing the evolving clinical picture, which notably included a progressive enhancement in the patient's EDSS scoring. Concurrently, a significant aspect of the therapeutic agenda involved tackling the complexities arising from lower urinary tract impairments. The primary emphasis lies in not only managing but also having a positive impact on the patient's daily life and well-being.

This case was initially presented by Papa et al. The authors highlighted novel findings previously undocumented in the medical literature: the hypointensities referred to as "black holes" seen in the T1 brain MRI scan after two months in both the bilateral middle cerebellar peduncles and internal capsule, along with marked sulci widening and spinal atrophy, were never reported before [7]. Herein, we aim to stress the importance of collaborative efforts among different medical specialties in enhancing the quality of life (QoL) for patients facing complex neurological conditions such as autoimmune GFAP.

## Conclusions

All in all, we conclude that a multifaceted treatment approach in patients with autoimmune GFAP astrocytopathy has favorable results regarding the improvement of QoL, and such complex cases should be referred ideally to specialized centers. Specialized neurourology centers, such as the one involved in our case, serve as ideal settings where multidisciplinary expertise converges to provide tailored care for individuals with intricate neurologic pathologies. They provide ideal settings to provide tailored care for patients with intricate neurologic pathologies.

Early neurourological intervention is critical in order to preserve the proper function of the lower urinary tract and avoid the use of an indwelling catheter. In that direction, invasive urodynamic tests are essential for the proper management of patients with autoimmune GFAP acrocytopathy. Last but not least, minimally invasive procedures along with more invasive options should always be offered to neurourological patients in specialized centers, allowing them to regain a high-level QoL while protecting the upper urinary tract at the same time.
